# Transfer of the presence seminar concept “Peer-support and Patient Competence” with participation of patients sharing their experience into a virtual teaching format

**DOI:** 10.3205/zma001371

**Published:** 2020-12-03

**Authors:** Andrea Kiemen, Theresa Baadte, Martina Jablotschkin, Joachim Weis

**Affiliations:** 1 Universitätsklinikum Freiburg, CCCF – ITZ, Stiftungsprofessur Selbsthilfeforschung, Freiburg, Germany

**Keywords:** peer-support, patient competence, empowerment, oncology, digital teaching, Covid-19

## Abstract

**Background:** In the innovative seminar “Peer-support and Patient Competence”, which was conceived as a face to face course, we teach various concepts of patient competence and invite patients to report about their illness experience and peer-group activities.

**Method:** Implementation of a face to face course into a virtual format via video conference.

**Result:** Despite concerns regarding the sensitive topic and technical challenges, the conversion of the seminar with interactive character, which was originally designed in a face-to-face format, into a virtual one was successful. Both lecturers and participants experienced the seminar as satisfactory.

**Conclusion:** In times of Covid-19, this virtual course experience can encourage colleagues to restructure their face to face seminars into innovative and virtual teaching formats.

## Zusammenfassung

**Hintergrund: **Das thematisch innovative und in Präsenzform konzipierte Seminar „Selbsthilfe und Patient*innenkompetenz“ vermittelt Konzepte der Patient*innenkompetenz, die u.a. durch die Schilderung persönlicher Krankheitserfahrung Betroffener veranschaulicht werden.

**Methode: **Umsetzung des Präsenz-Seminars in digitale Form via Video-Konferenz.

**Ergebnis: **Trotz Bedenken hinsichtlich der sensiblen Thematik und technischer Herausforderungen, ist die Umsetzung des ursprünglich in Präsenzform konzipierten Seminars in ein digitales Format mit interaktivem Charakter gelungen. Das Seminar wurde von Dozent*innen und Teilnehmer*innen gleichermaßen als zufriedenstellend erlebt.

**Schlussfolgerung:** In Zeiten von Covid-19 kann diese digitale Seminarerfahrung als Ermutigung für eine umstrukturierte Lehrveranstaltung dienen.

## Short description of the seminar

In the seminar “Peer-support and Patient Competence” of the Endowed Chair for Self-Help Research at the University Medical Center Freiburg, students of medicine, psychology and health education learn about peer-to-peer support as supplementary psychosocial support for chronically ill patients, aimed at the enhancement of patient competence and empowerment [1]. Representatives of local peer-to-peer / self-help organizations (SHOs; i.e., German synonym) are invited to share their individual experience of illness and their activities in the SHOs [2], [3]. SHO representatives report both on their individual coping resources and on the psychological distress experienced due to their diagnosis and treatment, such as anxiety, depression, worries, and fears. In addition, we teach various concepts of patient competence and specific aspects of coping with the illness (e.g. coping strategies, self-efficacy expectations [4], resilience [5], post-traumatic growth [6]) [7]. The seminar provides practical experience to the students by encountering with patients affected with various diseases and learning about the personal experiences. Especially these personal and intimate reports by patients represent a particular challenge for restructuring the course into a virtual format.

## Seminar preparation, challenges, and implementation

The seminar is a voluntary course not relevant for examination. Students can acquire 3 ECTS points as a course achievement for a topic presentation in the seminar or a term paper. The seminar is leaded by Prof Weis together with three colleagues. Developing the virtual format we intensively discussed different possibilities of online teaching formats for the seminar:

- PowerPoint voice-over presentations and guideline-based, recorded interviews with SHO representatives, which are uploaded to the ILIAS learning platform at the time of the seminar and are worked on by the students on their own;
- Live video conference together with the students and the SHO representatives bearing the risk of technical problems during the seminar and potential fear of usability and technical barriers of the elderly SHO representatives.

After intensive discussion and consideration in the team, we decided to use an interactive online live format. The e-learning team of the university Freiburg supported our decision-making process. 

Nevertheless, great efforts were needed to transfer the original face to face format into a virtual one, and to implement the new virtual course:

- Technical handling of the learning platform ILIAS as well as testing of different options for live conferences;
- Acquisition of new technical equipment (headsets, webcams, laptops);
- Creation of learning documents for students and SHO representatives as well as support of the ILIAS forum for students' questions;
- Didactic challenge: Motivating students over the duration of the seminar by means of suitable interactive methods in a relatively short preparation time;
- Conducting test sessions with the SHO representatives and technical support.

## Expectations of the teaching team

Our aim was to convey the teaching and learning contents and the specific interactive character of this seminar, which is characterized by individual experience reports of the SHO representatives sharing with the students in the online format. We asked ourselves how attentive the students in their home setting would be to the online event, and how they can engage in an interactive encounter with the patients. We were also curious to see how the SHO representatives succeed in communicating their experience of illness in the online format against the background of possible impairments, e.g. laryngectomy patients with speech aids. 

## Experience

The live format using the Zoom software has been proven to be successful. The 20 registered students were able to connect online without main problems of internet capacity. In only few cases some participants had problems with the video or sound function due to insufficient technical equipment or internet capacity. Nevertheless, we experienced an unexpected openness and active participation of the students. In addition, the students showed a high level of self-management.

The evaluation of the course via online survey showed that 90.9% of the students rated the “relevance of the topics covered” and the “teaching of the contents by the lecturers” as positive; likewise, the majority rated the ‘presentation of the peer-to-peer activities by the representatives of the SHOs’ overall as enriching. In summary, 58.8% of the students rated the seminar with grade 1-2 (ranging from 1=best to 6=worst), grade 3, as the worst result, was rated by 5.88%. In the last session of the seminar the students gave personal feedback and pointed out as positive aspects the interactive design of the seminar using the whiteboard, workout groups, and online surveys in which the results could be discussed promptly, as very good.

In the virtual format the SHO representatives were able to communicate their experiences with the disease, the psychosocial distress and the various support they received in an impressive and true-to-life way. Some of the students were touched and affected by the reports of the SHO representatives and after a slight hesitation, they dared to ask questions. 

## Outlook and transfer of the findings into further practice

Since online teaching is to be continued in Baden-Württemberg for the time being, we will expand and improve the use of interactive tools. Based on our experience, we will limit the number of participants to 30 students to ensure personal exchange among each other and with the SHO representatives. Improvement is needed especially in the technical support for the students on site, so that they can participate without any restrictions with video and sound function.

## Competing interests

The authors declare that they have no competing interests. 
